# Validity Evaluation of the Fitbit Charge2 and the Garmin vivosmart HR+ in Free-Living Environments in an Older Adult Cohort

**DOI:** 10.2196/13084

**Published:** 2019-06-19

**Authors:** Salvatore Tedesco, Marco Sica, Andrea Ancillao, Suzanne Timmons, John Barton, Brendan O'Flynn

**Affiliations:** 1 Tyndall National Institute, University College Cork Cork Ireland; 2 Centre for Gerontology and Rehabilitation, University College Cork Cork Ireland

**Keywords:** aging, fitness trackers, wristbands, older adults, wearable activity trackers, Fitbit, Garmin, energy expenditure, physical activity, sleep

## Abstract

**Background:**

Few studies have investigated the validity of mainstream wrist-based activity trackers in healthy older adults in real life, as opposed to laboratory settings.

**Objective:**

This study explored the performance of two wrist-worn trackers (Fitbit Charge 2 and Garmin vivosmart HR+) in estimating steps, energy expenditure, moderate-to-vigorous physical activity (MVPA) levels, and sleep parameters (total sleep time [TST] and wake after sleep onset [WASO]) against gold-standard technologies in a cohort of healthy older adults in a free-living environment.

**Methods:**

Overall, 20 participants (>65 years) took part in the study. The devices were worn by the participants for 24 hours, and the results were compared against validated technology (ActiGraph and New-Lifestyles NL-2000i). Mean error, mean percentage error (MPE), mean absolute percentage error (MAPE), intraclass correlation (ICC), and Bland-Altman plots were computed for all the parameters considered.

**Results:**

For step counting, all trackers were highly correlated with one another (ICCs>0.89). Although the Fitbit tended to overcount steps (MPE=12.36%), the Garmin and ActiGraph undercounted (MPE 9.36% and 11.53%, respectively). The Garmin had poor ICC values when energy expenditure was compared against the criterion. The Fitbit had moderate-to-good ICCs in comparison to the other activity trackers, and showed the best results (MAPE=12.25%), although it underestimated calories burned. For MVPA levels estimation, the wristband trackers were highly correlated (ICC=0.96); however, they were moderately correlated against the criterion and they overestimated MVPA activity minutes. For the sleep parameters, the ICCs were poor for all cases, except when comparing the Fitbit with the criterion, which showed moderate agreement. The TST was slightly overestimated with the Fitbit, although it provided good results with an average MAPE equal to 10.13%. Conversely, WASO estimation was poorer and was overestimated by the Fitbit but underestimated by the Garmin. Again, the Fitbit was the most accurate, with an average MAPE of 49.7%.

**Conclusions:**

The tested well-known devices could be adopted to estimate steps, energy expenditure, and sleep duration with an acceptable level of accuracy in the population of interest, although clinicians should be cautious in considering other parameters for clinical and research purposes.

## Introduction

Fitness trackers are popular devices used by athletes and the general public to monitor their physical activity levels, sport performance, and even their general health status in real time, with the latter having the potential to also predict the person’s future health status [1]. Compared to other body positions, the wrist has been identified as the most suitable location for enhancing user acceptability and the user-friendliness of the device [2]. Common consumer-level, wrist-worn devices typically provide data on step count, distance traveled, number of floors climbed, and minutes of physical activity, as well as sport-related activity recognition, physiological measurements, energy expenditure, and sleep patterns. This information can promote a healthier lifestyle or an optimal training program through user-friendly visual feedback of current status or performance compared to set targets.

Fitness trackers based on motion sensors are also being used for monitoring biomechanical quantities of clinical interest, such as gait analysis applications [3,4], indirect estimation of ground reaction forces, and posture in general [5,6].

Although the number of studies investigating the validity and reliability of different fitness trackers is growing, the majority of the evidence is limited to young and middle-aged adult populations, mostly in good health [7-9]. Considering the multiple applications of wrist-based technology and its potential adoption in health care, and with an aging population, it is important to investigate the use of these devices in different populations, such as older people [10]. Although older adults perceive commercial trackers as useful and acceptable [11,12], older person-specific activity trackers are still limited [13].

Few studies have investigated the validity of mainstream wrist-based activity trackers in healthy older adults [14,15]. However, such investigations mainly involved a protocol structured around a number of daily activities simulated or recreated in a laboratory environment. Studies that investigated fitness trackers’ performance when used by older people in their home environment, where older adults can perform their real daily routine, are scarce and mainly limited to step-counting features [16]. This study reviewed the validity and reliability of consumer-grade activity trackers in older community-dwelling adults through seven observational studies, of which only five studied free-living settings for a monitoring period of between 3 and 7 days.

For example, Paul et al [17] reported that the average steps per day measured over 7 days in a community-dwelling older adult population with a Fitbit and an ActiGraph showed excellent agreement, with the ActiGraph undercounting steps compared against participants’ physical activity logs.

In another study, a Fitbit Flex and an ActiGraph were worn by a cohort of cardiac patients and their family members to measure steps and moderate-to-vigorous physical activity (MVPA) levels for 4 days [18]. It showed a significant correlation for step counts but lower values for MVPA, with the Fitbit Flex slightly overestimating both parameters.

Boeselt et al [19] compared a Polar A300 with a BodyMedia SenseWear in a cohort of patients with chronic obstructive pulmonary disease (mean age 66.4 years). Participants used the devices for three consecutive days, measuring steps, calories burned, daily activity time, and metabolic equivalents. The study showed a high correlation for step count and calories burned.

Farina and Lowry [20] compared the accuracy of step counts from two consumer-level activity monitors (Misfit Shine on both wrist and waist, and Fitbit Charge HR on the wrist) against two waist-worn reference devices (ActiGraph GT3X+ and New Lifestyle NL2000i) in healthy, community-dwelling older adults in free-living conditions over seven consecutive days. All consumer-level activity monitors positively correlated with reference devices. Compared to the ActiGraph GT3X+, the waist-worn Misfit Shine displayed the highest agreement, whereas the wrist-worn devices showed poorer performances.

Finally, Burton et al [21] reported good reliability and validity for the Fitbit Flex and Fitbit Charge HR compared against a GENEactiv accelerometer in a free-living environment over 14 days. Step count, distance traveled, MVPA minutes, and sleep were measured. Good strength of agreement was found for total distance and steps (obtained with the fitness tracker) and the MVPA estimated by the GENEactiv.

It is evident that a comparative analysis of mainstream trackers worn by healthy older people in a more ecologically valid environment is needed. This study aims to investigate the reliability and accuracy of the wrist-based Fitbit Charge 2 and the Garmin vivosmart HR+ activity trackers in the estimation of daily step count, total calorie expenditure, MVPA, and sleep parameters within a home environment in a cohort of older adults.

## Methods

### Participants

This study was based on a sample of 20 healthy older people (9 males, 11 females). Volunteers were recruited via a general invitation email, posters, and word of mouth to exstaff at University College Cork (Cork, Ireland) and their relatives, and also through local social and voluntary groups that had older adults as members. They were informed of the study by the Centre for Gerontology and Rehabilitation in University College Cork.

For the cohort, the inclusion criteria were age 65 years and older, with no history of neurological or other disorders or disability that could affect the participant’s movements, and in good general health. Before participation, volunteers received an oral and written explanation of the study protocol, and written consent was obtained. Sociodemographic information was collected on gender, age, weight, height, and dominant arm. The study received approval by the Clinical Research Ethics Committee at the University College Cork. Demographic information on the participants who completed the study protocol is presented in Table 1.

**Table 1:** Participant characteristics (N=20).

Characteristic	Males (n=9)	Females (n=11)
Age (years), mean (SD)	70.2 (2.9)	71.1 (3.1)
Height (cm), mean (SD)	176.8 (4.7)	161.1 (6.9)
Weight (kg), mean (SD)	81.6 (12.5)	65.9 (9.3)
People with right dominant arm, n	9	10
People with left dominant arm, n	0	1

### Equipment

The following consumer-level and research-grade devices were selected for comparison:

1. Fitbit Charge 2 (Fitbit Inc, San Francisco, CA, USA): a wrist-based device with a large organic light-emitting diode screen featuring heartrate monitoring and tracking of steps, distance, calories burned, floors climbed, active minutes, and sleep duration.
2. Garmin vivosmart HR+ (Garmin, Olathe, KS, USA): a wrist-based device that monitors heart rate, calories burned, intensity of fitness activities, distance, time, and pace for indoor or outdoor activities.
3. ActiGraph GT9X-BT (ActiGraph LLC, Pensacola, FL, USA): a research-grade activity monitor based on motion sensors that provide raw data and numerous activity and sleep measures (eg, activity counts, energy expenditure, steps taken, activity/sedentary bouts, sleep latency/efficiency) via publicly available validated algorithms. The device can be worn on the waist, hip, wrist, ankle, or thigh.
4. New-Lifestyles NL-2000i Activity Monitor (New-Lifestyles Inc, Lee’s Summit, MO, USA): a research-grade measurement tool for data collection based on a three-dimensional piezoelectric accelerometer. The device can provide details on steps, MVPA, total/active calories, and distance, and can store data for 7 to 14 days. Examples of studies that have validated the pedometer are available [20,22-24].

### Experimental Protocol

**Figure 1:**
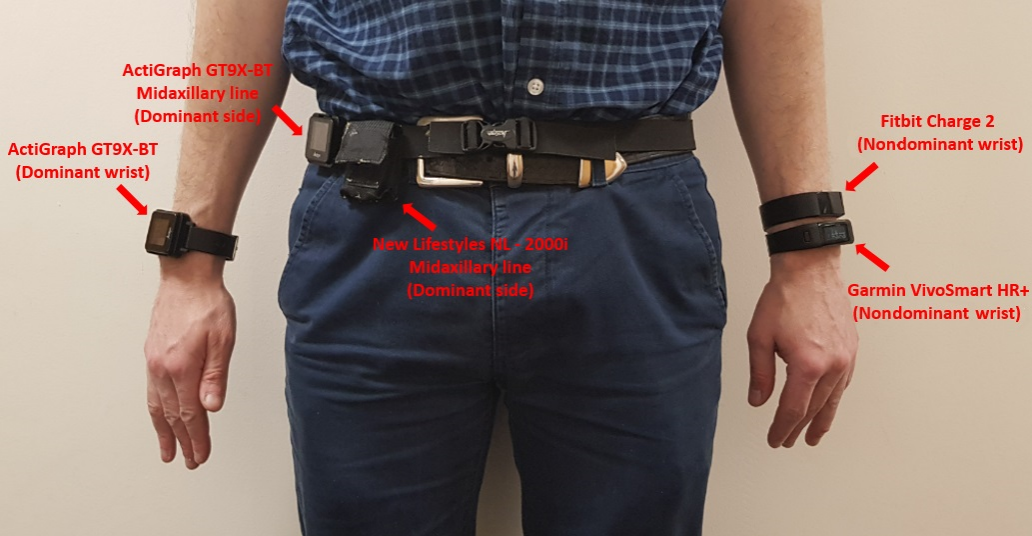
Placement of devices on a participant.

Two consumer-based wrist-mounted brands were tested (Fitbit and Garmin), worn on the nondominant arm. The trackers’ position on the wrist was randomized. The dominant side of the waist (midaxillary line) and the dominant wrist are reported to be optimal for monitoring energy expenditure, MVPA, and sleep in older adults [25-28]; therefore, two ActiGraph monitors were located in these positions as a reference for those parameters. Energy expenditure, MVPA levels, and steps were measured with the ActiGraph on the waist; sleep parameters were extrapolated from the ActiGraph on the wrist. The New-Lifestyles NL-2000i tracker was also worn on the dominant waist (midaxillary line) and was considered as a reference for step counting. Figure 1 illustrates the body positions of the different devices on a participant.

The algorithm adopted by the ActiGraph for estimating energy expenditure was based on the method designed by Crouter et al [29] and also considered in Patterson et al [26]. This method provides estimations expressed in metabolic equivalent (MET), which are later converted into total calories per day. Likewise, the algorithms of Troiano et al [30] and Cole-Kripke et al [31] were considered for estimating MVPA levels and sleep parameters, respectively, through the ActiGraph accelerometer [28,32]. By definition, MVPA level is the amount of time spent performing any activities requiring more than 3 METs, which according to Troiano et al [30] is defined by the ActiGraph by at least 2020 counts per minute. Finally, to guarantee a fair comparison for the different trackers, only the sleep parameters measured by both the Fitbit and Garmin were analyzed. Those parameters were the total sleep time (TST), and the wake after sleep onset (WASO). Participants were asked to complete a sleep diary as well, and the in-bed and out-bed information required by the Cole-Kripke method were input manually according to the values reported in the sleep diary.

The devices were attached on to the person in the morning for data collection and were returned to the researchers the following morning. All trackers were removed by the participants during bathing, whereas only the trackers on the waist were removed during sleep.

Nonwear periods were defined as 90 minutes or more with no activity counts [33]. A valid day was defined as 10 wearing hours or more in a 24-hour period [30].

### Statistical Analysis

Descriptive statistics were run on the computed parameters. The following indicators were computed for each parameter and device: mean estimated value with related standard deviation (SD), mean bias with standard deviation, mean percentage error (MPE) with standard deviation, and mean absolute percentage error (MAPE). Intraclass correlation (ICC[2,1]) was performed for each tracker compared against all other devices and the criterion as well. The related 95% confidence intervals (CIs) were also computed. ICC values less than 0.5, between 0.5 and 0.75, between 0.75 and 0.9, and greater than 0.90 are indicative of poor, moderate, good, and excellent reliability, respectively [34]. Bland-Altman plots were also obtained for every parameter comparing all the possible permutations of trackers and the criterion. All statistical analyses were performed using MATLAB (MathWorks, Natick, MA, USA).

## Results

Overall, 20 participants took part in the data collection. All participants were white of Irish and British ancestry. Data collection was carried out at the Tyndall National Institute between April 2018 and August 2018.

**Figure 2:**
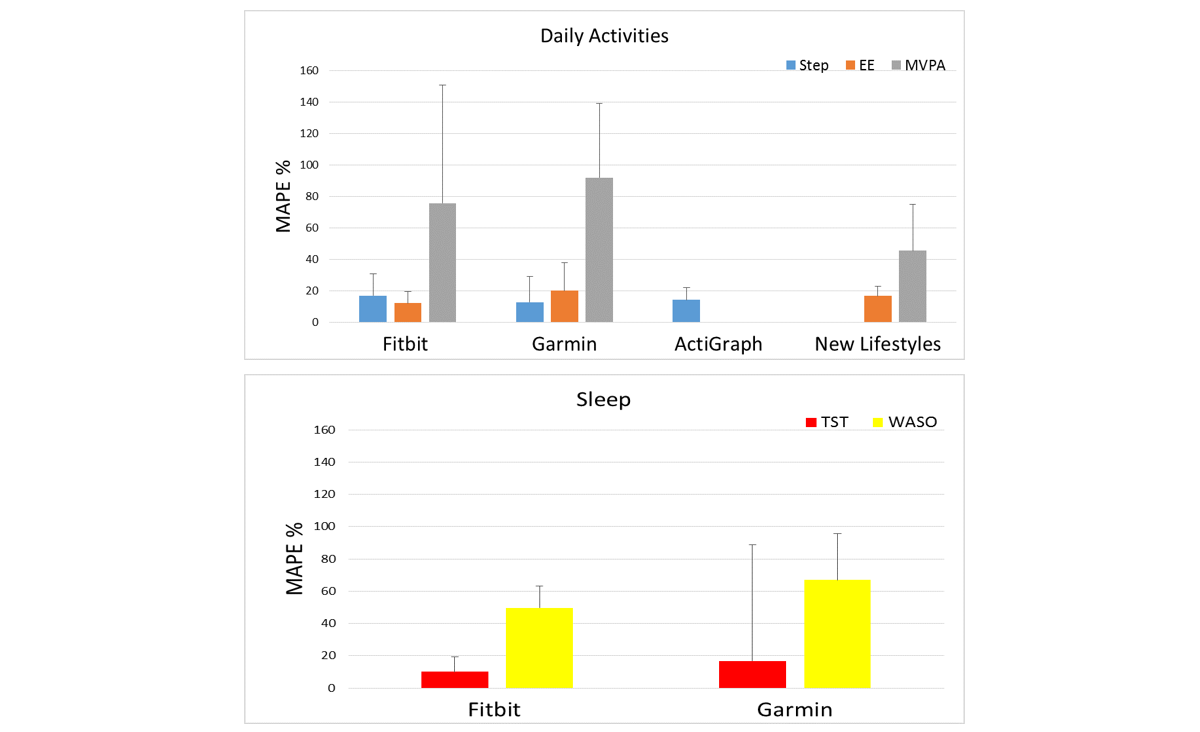
Mean absolute percentage error (MAPE) with standard deviation for each parameter and tracker. EE: energy expenditure; MVPA: moderate-to-vigorous physical activity; TST: total sleep time; WASO: wake after sleep onset.

Table 2 shows the mean values measured, mean error, mean percentage error, and MAPE with related standard deviations for the activity monitors and each parameter. Likewise, Table 3 shows the related ICCs with the 95% CI for each tracker and every parameter. Figure 2 displays the MAPE.

The average wear time for each tracker among all participants was mean 963 (SD 102) minutes per day; thus, every monitored day for each participant was deemed a valid test day above the 10 wearing hours threshold. All monitored days were on weekdays.

For step counting, all the trackers were highly correlated with one another (ICCs>0.89). Although the Fitbit tended to overcount steps (MPE=12.36%), the Garmin and ActiGraph undercounted with a MPE of 9.36% and 11.53%, respectively. For the MAPE, the Garmin and ActiGraph were slightly more accurate with mean MAPEs of 12.89% and 14.23%, respectively. Therefore, all the considered activity trackers can accurately capture steps when worn on the nondominant wrist.

However, the Garmin had poor ICC values when comparing energy expenditure against the New-Lifestyles NL-2000i and the criterion. Likewise, similar results were shown when comparing the New-Lifestyles NL-2000i and the criterion. Conversely, the Fitbit had moderate-to-good ICCs compared against the other activity trackers. All the tested activity monitors underestimated the amount of calories burned, with the Fitbit showing the best results with a MAPE of 12.25%.

For MVPA level estimation, the Fitbit and Garmin were highly correlated (ICC=0.96), whereas the New-Lifestyles NL-2000i showed poor correlation with these two devices. However, all monitors were moderately correlated against the criterion. The Fitbit and Garmin overestimated the minutes of MVPA activity (mean 12.63, SD 28.31 and mean 13.8, SD 35.7 minutes per day), whereas New-Lifestyles-2000i underestimated, although it showed the best MAPE results, at 45.45%, suggesting that consumer-grade activity trackers may not be reliable in estimating MVPA in older adults.

When analyzing the sleep parameters, the ICCs were poor for all cases, except when comparing the Fitbit to the criterion, which showed a moderate agreement. The TST was slightly overestimated with the Fitbit (mean 5.72, SD 49.11 minutes), although it provided good results with a mean MAPE equal to 10.13%. Conversely, the WASO estimation was poorer; it was overestimated by the Fitbit but underestimated by the Garmin. Again, the Fitbit was the most accurate, with a mean MAPE of 49.7%.

**Figure 3:**
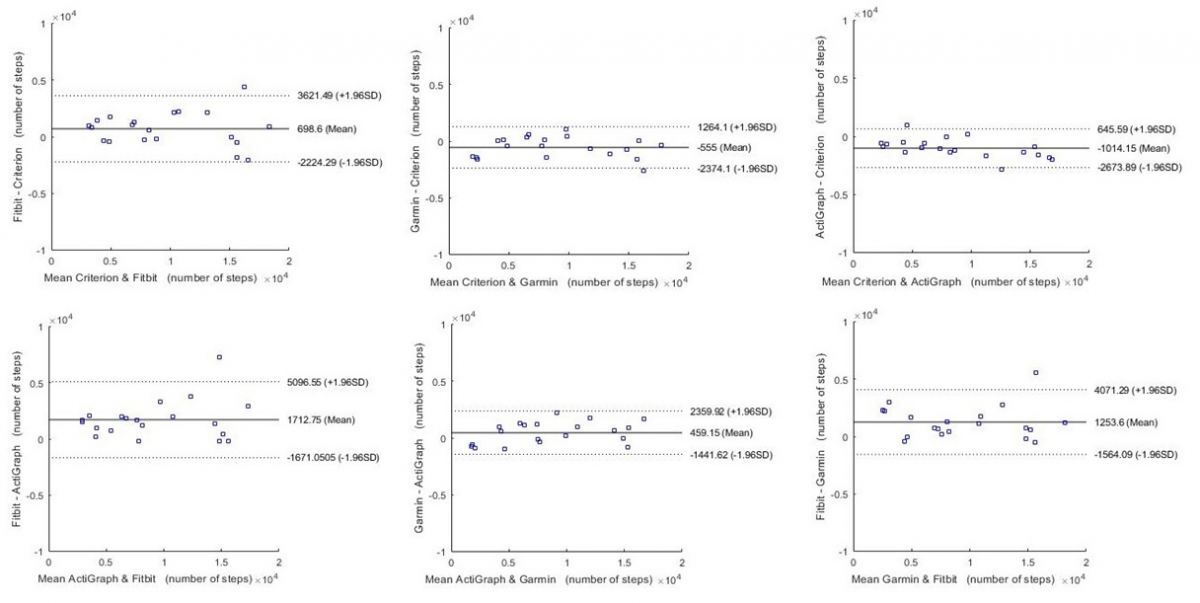
Bland-Altman plots for steps.

**Figure 4:**
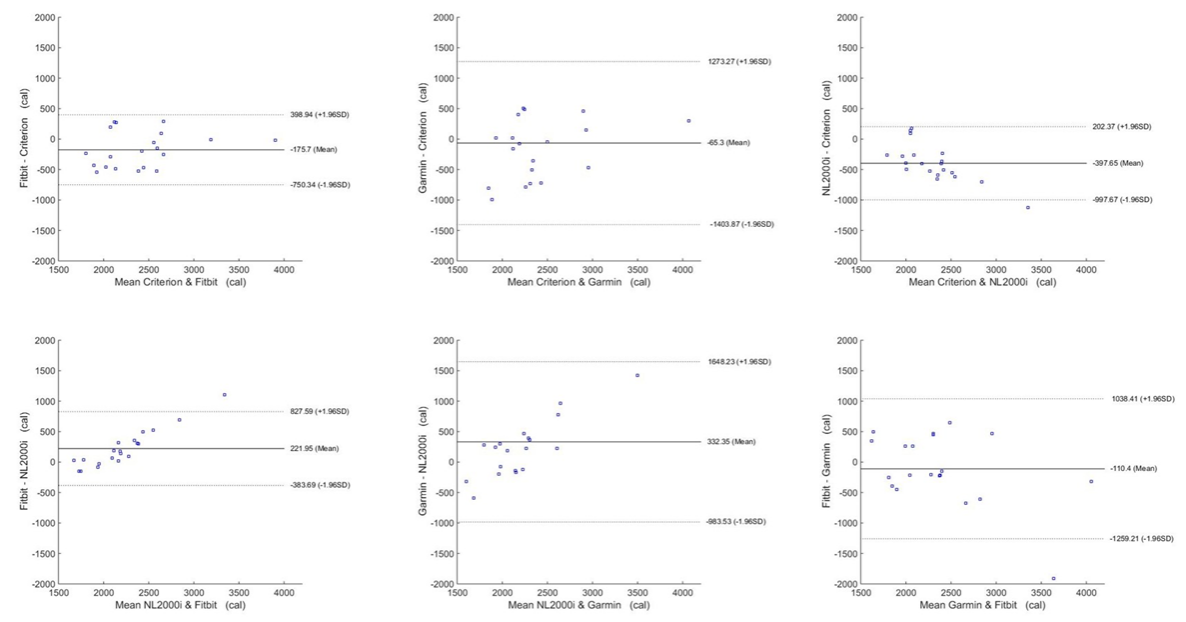
Bland-Altman plots for energy expenditure.

**Figure 5:**
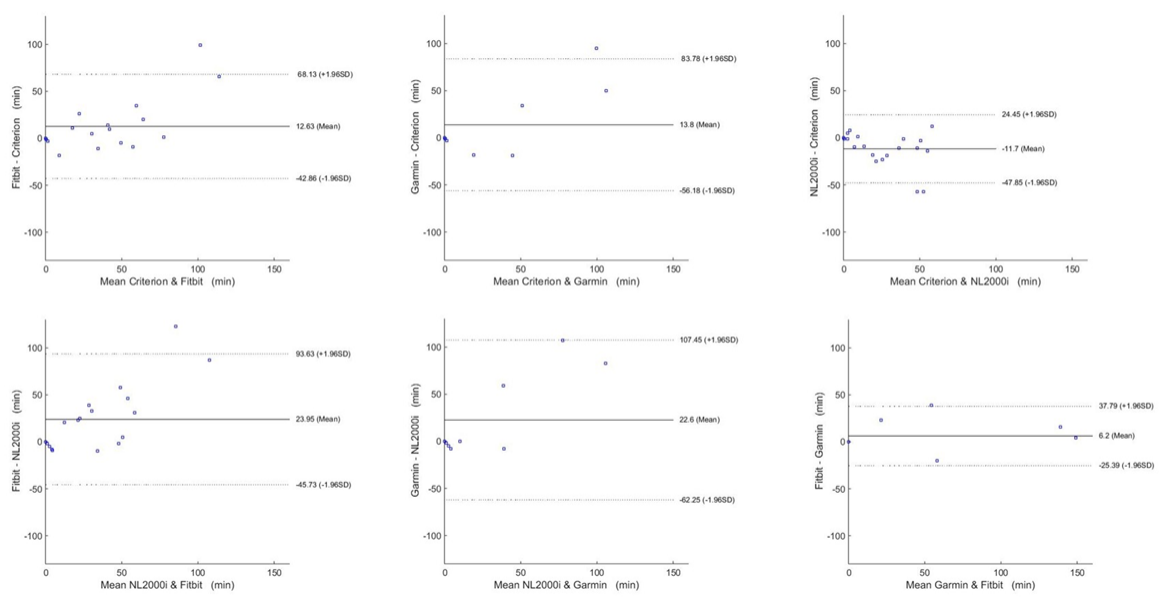
Bland-Altman plots for moderate-to-vigorous physical activity.

**Figure 6:**
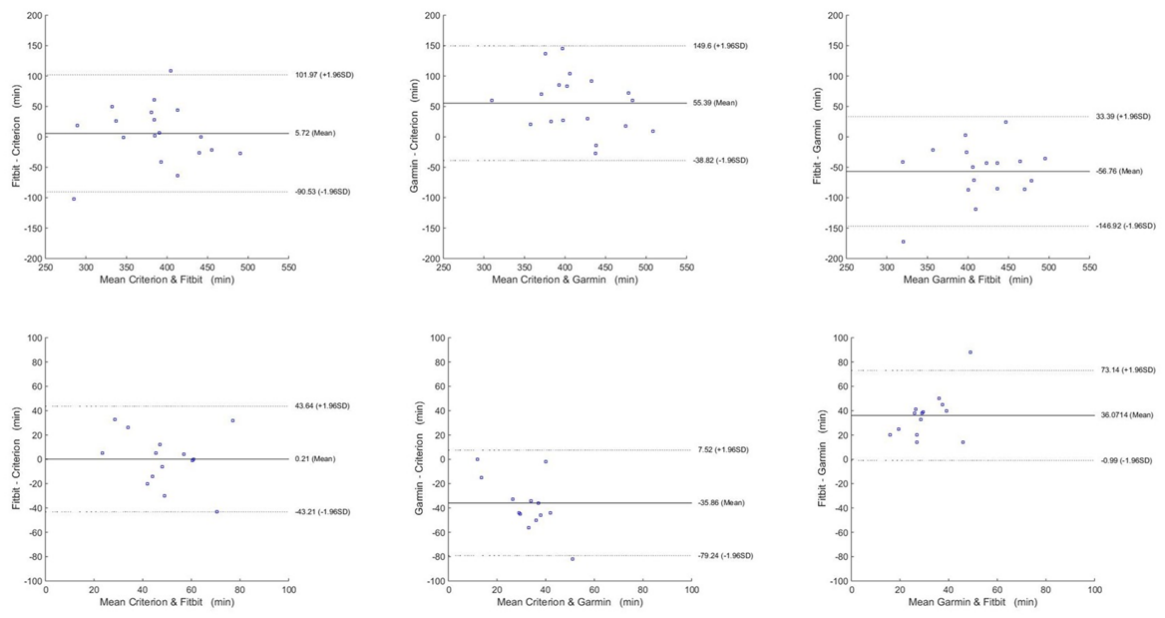
Bland-Altman plots for sleep parameters. Top row: total sleep time; bottom row: wake after sleep onset.

The Bland-Altman plots are shown in Figures 3-6 for steps, energy expenditure, MVPA, and sleep parameters, respectively, and summarized in Table 4.

**Table 2:** Mean values measured, mean error, mean percentagage error, and mean absolute percentage error with related standard deviation for each parameter and tracker (N=20).

Parameter		Mean (SD)	Mean error (SD)	MPE^a^ (SD)	MAPE^b^ (SD)
**Steps**					
	Fitbit	10088.45 (5067.32)	698.60 (1491.27)	12.36 (18.26)	17.05 (13.71)
	Garmin	8834.85 (5067.07)	–555.00 (928.11)	–9.36 (18.55)	12.90 (16.16)
	ActiGraph	8375.70 (4716.39)	–1014.15 (846.81)	–11.53 (11.55)	14.23 (7.76)
**Energy expenditure (cal)**					
	Fitbit	2324.25 (547.02)	–175.70 (293.18)	–6.98 (12.67)	12.26 (7.33)
	Garmin	2434.65 (804.12)	–65.30 (682.94)	–2.09 (27.27)	20.05 (18.04)
	NL2000i	2102.30 (256.93)	–397.65 (306.13)	–14.61 (10.41)	16.70 (6.3)
**MVPA^c^ (min)**					
	Fitbit	44.32 (45.83)	12.63 (28.31)	31.78 (103.51)	75.74 (75.32)
	Garmin	39.10 (57.18)	13.80 (35.70)	6.42 (109.75)	91.98 (47.13)
	NL2000i	20.30 (19.03)	–11.70 (18.45)	–41.43 (35.37)	45.45 (29.67)
**TST^d^ (min)**					
	Fitbit	389.83 (59.33)	5.72 (49.11)	2.27 (13.66)	10.14 (9.12)
	Garmin	442.83 (48.64)	55.39 (48.07)	15.85 (14.56)	16.86 (13.3)
**WASO^e^ (min)**					
	Fitbit	49.21 (16.5)	0.21 (22.15)	25.02 (84.72)	49.73 (72.03)
	Garmin	13.14 (8.93)	–35.86 (22.13)	–66.89 (28.61)	66.89 (28.61)

^a^MPE: mean percentage error.

^b^MAPE: mean absolute percentage error.

^c^MVPA: moderate-to-vigorous physical activity.

^d^TST: total sleep time.

^e^WASO: wake after sleep onset.

**Table 3:** Intraclass correlation (ICC) and 95% CI for each parameter and tracker (N=20).

Parameter		ICC (95% CI)				
		Fitbit	Garmin	ActiGraph (waist)	NL2000i	Criterion
**Step**						
	Fitbit	—^a^	0.93 (0.65, 0.98)	0.89 (0.39, 0.97)	—	0.95 (0.87, 0.98)
	Garmin	—	—	0.98 (0.94, 0.99)	—	0.98 (0.93, 0.99)
	ActiGraph (waist)	—	—	—	—	0.97 (0.59, 0.99)
**Energy expenditure**						
	Fitbit	—	0.64 (0.29, 0.84)	—	0.66 (0.21, 0.86)	0.80 (0.48, 0.92)
	Garmin	—	—	—	0.32 (–0.07, 0.65)	0.48 (0.05, 0.76)
	NL2000i	—	—	—	—	0.45 (–0.10, 0.78)
**MVPA^b^**						
	Fitbit	—	0.96 (0.86, 0.99)	—	0.41 (–0.01, 0.72)	0.69 (0.35, 0.87)
	Garmin	—	—		0.46 (–0.11, 0.82)	0.68 (0.18, 0.91)
	NL2000i	—	—	—	—	0.60 (0.18, 0.83)
**TST^c^**						
	Fitbit	—	0.43 (–0.10, 0.77)	—	—	0.67 (0.30, 0.86)
	Garmin	—	—	—	—	0.42 (–0.10, 0.76)
**WASO^d^**						
	Fitbit	—	<0.01 (–0.08, 0.18)	—	—	0.32 (–0.28, 0.72)
	Garmin	—	—	—	—	0.01 (–0.10, 0.25)

^a^Not applicable.

^b^MVPA: moderate-to-vigorous physical activity.

^c^TST: total sleep time.

^d^WASO: wake after sleep onset.

**Table 4:** Bland-Altman plots summary for each parameter and tracker (N=20).

Parameter			Fitbit	Garmin	ActiGraph (waist)	NL2000i	Criterion
**Step**							
	**Fitbit**						
		Mean error (SD)	—^a^	1253.60 (1437.60)	1712.75 (1726.43)	—	698.60 (1491.27)
		95% LoA^b^	—	–1564.09, 4071.29	–1671.05, 5096.55	—	–2224.29, 3621.49
	**Garmin**						
		Mean error (SD)	—	—	459.15 (969.78)	—	–555 (928.11)
		95% LoA	—	—	–1441.62, 2359.92	—	–2374.10, 1264.10
	**ActiGraph (waist)**						
		Mean error (SD)	—	—	—	—	–1014.15 (846.81)
		95% LoA	—	—	—	—	–2673.89, 645.59
**Energy expenditure (cal)**							
	**Fitbit**						
		Mean error (SD)	—	–110.40 (586.13)	—	221.95 (309.00)	–175.7 (293.18)
		95% LoA	—	–1259.21, 1038.41	—	–383.69, 827.59	–750.34, 398.94
	**Garmin**						
		Mean error (SD)	—	—	—	332.35 (671.37)	–65.3 (682.94)
		95% LoA	—	—	—	–983.53, 1648.23	–1403.87, 1273.27
	**NL2000i**						
		Mean error (SD)	—	—	—	—	–397.65 (306.13)
		95% LoA	—	—	—	—	–997.67, 202.37
**MVPA^c^ (min)**							
		Fitbit					
		Mean error (SD)	—	6.20 (16.12)	—	23.95 (35.55)	12.63 (28.31)
		95% LoA	—	–25.39, 37.79	—	–45.73, 93.63	–42.86, 68.13
	**Garmin**						
		Mean error (SD)	—	—	—	22.60 (43.29)	13.80 (35.70)
		95% LoA	—	—		–62.25, 107.45	–56.18, 83.78
	**NL2000i**						
		Mean error (SD)	—	—	—	—	–11.70 (18.45)
		95% LoA	—	—	—	—	–47.85, 24.45
**TST^d^ (min)**							
	**Fitbit**						
		Mean error (SD)	—	–56.76 (45.99)	—	—	5.72 (49.11)
		95% LoA	—	–146.92, 33.39	—	—	–90.53, 101.97
	**Garmin**						
		Mean error (SD)	—	—	—	—	55.39 (48.07)
		95% LoA	—	—	—	—	–38.82, 149.60
**WASO^e^ (min)**							
	**Fitbit**						
		Mean error (SD)	—	36.07 (18.91)	—	—	0.21 (22.15)
		95% LoA	—	–0.99, 73.14	—	—	–43.21, 43.64
	**Garmin**						
		Mean error (SD)	—	—	—	—	–35.86 (22.13)
		95% LoA	—	—	—	—	–79.24, 7.52

^a^Not applicable.

^b^LoA: limits of agreement.

^c^MVPA: moderate-to-vigorous physical activity.

^d^TST: total sleep time.

^e^WASO: wake after sleep onset.

## Discussion

This investigation is one of the first studies to investigate the reliability and accuracy of two consumer-level, wrist-based activity trackers in the estimation of daily step count, total calories expenditure, MVPA, and sleep parameters in a home environment for a cohort of healthy older adults.

Results show that the mainstream monitors may be adopted to estimate steps, energy expenditure, and some sleep parameters (eg, TST) with a certain level of accuracy in a healthy older adult population in free-living settings, whereas other variables (MVPA and WASO) may show excessively large errors.

The measured mean values are largely consistent with data reported studying the same population of interest in other studies adopting non-consumer-level technologies for energy expenditure [26], steps and MVPA [35], and sleep analysis [36].

Regarding step-counting performance, all the trackers presented a good strength of agreement among one another and against the reference device. Also, this study confirms previous findings [17,18] indicating a slight overcounting by the Fitbit device and an undercounting by the ActiGraph. In absolute terms, as shown by the MAPE, there is no significant difference between the Fitbit and the Garmin monitors when monitoring steps.

Similar considerations could be drawn for energy expenditure; however, only the Fitbit shows moderate-to-good agreement with the other trackers. The Fitbit underestimated energy expenditure in our study, confirming findings illustrated in the review by Feehan et al [37], which cited a number of studies in which Fitbits worn on the wrist in free-living settings slightly underestimated METs by 7% (when compared against doubly labeled water), and showed a −10% measurement error (against the SenseWear), and provided MAPE values varying from 16% to 30% when compared with measurements from an ActiGraph or Actiheart accelerometer. However, most of the studies reviewed considered healthy adults and not older adults; thus, the lower MAPE values reported in our study (mean 12.25%, SD 7.33%) may be due to the generally limited amount of moderate-to-vigorous activity performed by older adults. Fitbit showed the narrowest limit of agreement among the trackers, which indicated the device could underestimate the amount of calories per day up to 750.34 kcal and overestimate up to 398.94 kcal.

Due to its many health benefits reported, MVPA may represent an important aspect in people’s life and, with aging, this may become even more useful to guarantee independent living and prevention of noncommunicable diseases [35]. The current national MVPA recommendations consider a threshold of 30 minutes per day or 150 minutes per week. Therefore, a correct and reliable estimation of MVPA bouts helps support behavior change techniques applied to sedentary older adults. All the trackers considered (Fitbit, Garmin on the wrist, and New-Lifestyles NL-2000i on the waist) were moderately correlated with the reference. However, the Fitbit and Garmin showed an excellent strength of agreement between each other. The Fitbit and Garmin tended to overestimate MVPA with an average error of 12.63 minutes per day and 13.8 minutes per day, consistent with previously reported results [18], whereas the New-Lifestyles NL-2000i underestimated by 11.7 minutes per day. However, due to the limited moderate-to-vigorous activities performed, MAPE values are large, especially for the wrist-worn devices. The MAPE was mean 75.73% (SD 75.31%) and mean 91.98% (SD 47.13%) for the Fitbit and Garmin, respectively, confirming the large overestimation errors observed in Fitbit devices estimating MVPA in free-living settings compared with an ActiGraph accelerometer in healthy young adults and older adults living with a variety of chronic diseases (MAPEs >30%) [37]. The waist-worn device showed slightly better results both in terms of MAPE and limits of agreement (–47.85 to 24.45).

Finally, aging also impacts sleep, and changes occur in sleep patterns with aging (for example, decrease in the amount of slow wave sleep, increases in non-rapid eye movement sleep, increase in the number of spontaneous arousals, changes in the normal circadian sleep cycle) [38]. Moreover, older adults are more prone to develop sleep-related respiratory disorders, which are associated with cardiovascular disease, metabolic disorders, and impaired neurocognition [38]. Thus, low-cost, unobtrusive, and effective sleep monitoring devices such as consumer-level activity trackers are ideal for providing insightful details on the normal changes in sleeping patterns with advancing age. Between the Fitbit and the Garmin, the Fitbit was moderately correlated with the ActiGraph worn on the wrist, and only for the estimation of the sleeping time. TST was overestimated by a mean 5.72 minutes per day with a MAPE equal to 10.13% (SD 9.12%), which are largely consistent with findings reported in other studies adopting Fitbit devices to investigate sleep measurement accuracy in healthy young adults in free-living settings (MAPE approximately 10%) [37]. The Garmin showed larger errors with a MAPE of 16.8% (SD 13.3%). In contrast, WASO measurements were poorly correlated against the ActiGraph for both devices. Although the lowest mean error was 0.21 minutes per day for the Fitbit, MAPE was large (mean 49.7%, SD 72%) due to the generally limited amount of time spent awake overnight. Conversely, the Garmin significantly underestimated the measurements. Limits of agreement were similar for both trackers for both parameters. These performances may not be suitable for clinical-grade investigations because they require accurate measurements for supporting the decision-making process. For example, WASO is typically adopted as a criterion for discriminating insomnia and normal-sleeper groups (the general threshold is WASO ≥31 minutes per day occurring at least three times per week for at least 6 months) [39]; thus, the WASO estimation inaccuracy may hinder the adoption of mainstream wristband devices for clinical assessments in populations expected to have abnormal sleep patterns [40].

It is worth clarifying that there is no universally accepted definition of an acceptable degree of error for physical activity wearable devices. Some studies recommend that an acceptable measurement error under controlled conditions or for research purposes is within ±3% [41,42] and under free-living conditions is within ±10% [41,42]. Other studies recommend that mean errors of less than 20% have acceptable validity for clinical purposes [43]. This investigation considers the validity criteria between the tested and criterion physical activity measures for clinical purposes when the mean error is less than 20%. Results suggest that the tested devices could be adopted to estimate steps, energy expenditure, and sleep duration with an acceptable level of accuracy in the population of interest, whereas clinicians should be cautious in considering other parameters (eg, MVPA, awakenings) for clinical and research purposes. Although performance estimation is modest in some variables, it may still be adequate for guidance purposes. For instance, the ever-growing acceptance of wearable technologies by older people may push the adoption of wrist-worn trackers in behavior change investigations [11,44].

This study was limited to healthy older adults. As a consequence, it is difficult to indicate if these findings are generalizable to less active older adults or impaired or hospitalized older adults. Indeed, as shown in the literature, step-counting accuracy in people using a walking aid in a laboratory-structured protocol represents a challenge for all consumer-level trackers as evidenced by large MAPE values. Moreover, the small number of studied participants and the reduced intervention duration may also limit the generalizability of these findings. Thus, further studies would be needed to investigate activity trackers’ performance in a large cohort and also in nonhealthy populations.

Although the most common commercial trackers were considered in this study, it is difficult to indicate if results may translate to other consumer monitors on the market, due to the different algorithms they may employ.

This analysis was limited to some health parameters, whereas other variables, which may be of interest in older adults, could not be taken into account due to the lack of a gold-standard for nonlaboratory settings. Some examples are sedentary bouts, light activity bouts, the amount of time spent in different postures, distance traveled, speed, additional sleep measures (eg, sleep efficiency, sleep latency), and physiological measures, such as continuous heart rate measurements, blood oxygen saturation levels, galvanic skin response, blood pressure, or photoplethysmography, and these should be further investigated in future studies.

This study explored the performance of two wrist-worn trackers (Fitbit Charge2 and Garmin vivosmart HR+) estimating steps, energy expenditure, MVPA levels, and sleep parameters against gold-standard technologies in a free-living environment in a cohort of healthy participants aged 65 years and older.

This study confirmed that the wrist-worn devices are effective in estimating steps, energy expenditure, and some sleep parameters with a certain level of accuracy in healthy older adults (lower MAPE values: 12.89% for step counting with the Garmin, 12.25% for energy expenditure with the Fitbit, and 10.13% for TST estimation with the Fitbit). The results were coherent with previous studies, and the observed accuracy was acceptable for monitoring everyday activities. However, clinicians should be cautious in considering other parameters (eg, MVPA levels and WASO) for clinical and research purposes.

## Abbreviations

MAPE: mean absolute percentage error
MVPA: moderate-to-vigorous physical activity
TST: total sleep time
WASO: wake after sleep onset
ICC: intraclass correlation
MET: metabolic equivalent
